# Investigating Runner’s High: Changes in Mood and Endocannabinoid Concentrations after a 60 min Outdoor Run Considering Sex, Running Frequency, and Age

**DOI:** 10.3390/sports12090232

**Published:** 2024-08-28

**Authors:** Theresia Weiermair, Eva Svehlikova, Beate Boulgaropoulos, Christoph Magnes, Anita Eberl

**Affiliations:** 1 Institute for Biomedical Research and Technologies (HEALTH), Joanneum Research Forschungsgesellschaft m.b.H, Neue Stiftingtalstrasse 2, 8010 Graz, Austria; weiermair.theresia@outlook.com (T.W.);; 2 Division of Endocrinology and Diabetology, Department of Internal Medicine, Medical University of Graz, Auenbruggerplatz 15, 8036 Graz, Austria

**Keywords:** endocannabinoid, runner’s high, mood, exercise, endurance run

## Abstract

Runner’s high is a euphoric emotional state occurring during and post-physical exercise. Although previous data indicate endocannabinoids’ involvement in animal runner’s high, their role in human runner’s high remains to be established. We investigated runner’s high in healthy humans assessing mood and plasma endocannabinoid concentration changes pre- and post a 60 min outdoor run, considering sex (8 females/8 males), running frequency (4 occasional/12 regular runners) and age (median split 36 years). Mood, AEA, and 2-AG concentrations were significantly increased post-run considering all participants (*p* < 0.0001, *p* < 0.0001, *p* < 0.01, respectively), with 2-AG varying more than AEA concentrations. Concentrations of both endocannabinoids increased pre- to post-run in women (*p* < 0.01) but the AEA concentration increase was higher in females than in males (*p* < 0.05). Post-run concentration increase appeared to be more pronounced in occasional than in regular runners for 2-AG but not for AEA. However, regular runners experienced stronger mood increases and better post-run mood than occasional runners. Post-run endocannabinoid concentrations were increased regardless of age. AEA concentrations and their post-run changes were less affected by running frequency and age than those of 2-AG. These findings provide insights into the interplay of physical exercise, physiological/psychological factors and demographics, laying a valuable foundation for future research.

## 1. Introduction

Runner’s high is an emotional state during or after physical exercise characterized by reduced anxiety, pain sensitivity, and euphoria [1]. Endocannabinoids are believed to modulate stress-related endocrine and behavioral responses [2] and to influence mood [3] and they might play a relevant role in runner’s high [1,3,4].

The two endocannabinoids arachidonoyl ethanolamide (anandamide, AEA) and 2-arachidonoylglycerol (2-AG), which can cross the blood–brain barrier due to their high lipophilicity and thus bind to cannabinoid receptors in the brain [5,6,7,8], are biomarkers that can illustrate the physiological and psychological effects of runner’s high. AEA and 2-AG have different synthesis and degradation pathways [8] and also different potencies with regard to their main receptors: AEA acts as partial agonist of the cannabinoid receptor type 1, while 2-AG can bind to both cannabinoid receptor type 1 and 2 as a full agonist [9,10,11].

Although the driving neurobiological mechanisms of runner’s high have not yet been elucidated, data from preclinical studies suggest that endocannabinoids are strongly involved in its physiological pathways [8,12,13,14].

Neurobiological mechanisms are usually best studied in animal models, but animal models have a very limited use for studying the euphoric and mood-influencing dimensions of runner’s high, because mood assessment is meaningful to a very limited extent in animals. Therefore, studies investigating mood and AEA and/or 2-AG concentrations in parallel have been conducted in humans. These studies have assessed mood and simultaneously analyzed AEA and/or 2-AG concentrations from plasma pre- and post-physical exercise (e.g., running or cycling under laboratory conditions) [7,15,16,17,18,19]. A positive correlation between mood and endocannabinoid concentrations has been observed during and post physical exercise [7,16,18,20], and also increased AEA concentrations in post- compared to pre-physical exercise have been found [7,17,18,20]. In contrast, the observed effects of physical exercise on 2-AG concentrations have been inconsistent [1]. While all physical exercises in these earlier studies have taken place indoors, i.e., in a highly standardized environment, a more recent study has investigated endocannabinoid concentration changes after hiking under field conditions [21]. Results from this recent study have shown increased AEA and 2-AG concentrations post-physical exercise, but no correlation between endocannabinoid concentration changes and mood changes post-physical exercise was found [21]. Despite these available data, the role of endocannabinoids in human runner’s high after outdoor physical exercise is still not established.

Our study thus aimed to assess mood and concentrations of the endocannabinoids AEA and 2-AG in human plasma samples and their alterations pre- to post-run taking demographic characteristics (namely sex and age) and running frequency into account.

## 2. Materials and Methods

### 2.1. Study Procedures and Participants

This non-pharmacological interventional study was conducted in accordance with the Declaration of Helsinki and ICH guidelines for Good Clinical Practice [22]. The participants were advised to perform a 60 min outdoor run [23,24] and they were instructed to run as steady a pace as possible as endocannabinoid signaling has been shown to be dependent on the intensity of physical exercise [17,25]. All participants gave written informed consent before any study-related activities were started. In total, 16 healthy participants between 18 and 65 years were included in the clinical study (8 females and 8 males, 4 occasional and 12 regular runners, 8 participants < 36 years and 8 participants > 36 years). The regular runners who were performing an endurance run two or more times per week comprised seven females, five males, five participants < 36 years and seven participants > 36 years of age. The occasional runners’ subgroup (performing one or less endurance runs per week) consisted of one female, three males, three participants < 36 years and one participant > 36 years of age.

The participants were included on the basis of a physical examination and normal medical history. Exclusion criteria comprised general alcohol abuse or recent alcohol intake, cardiovascular or chronic diseases, acute illness, recent medication use, recent food intake (within 3 h before the study visit), drug use within the past 12 weeks, and pregnancy, breastfeeding, or intention to become pregnant.

### 2.2. Blood Sampling

Approximately 3 mL of venous blood were collected by a physician from the forearm of each participant pre- and immediately post-run. The blood was collected in a Vacuette^®^ K3E K3EDTA tube (Greiner Bio-One, Frickenhausen, Germany). Directly after collection, the blood was centrifuged for 10 min at 2000× *g* and 4 °C (Heraeus Multifuge X3R, Thermo Fisher Scientific, Waltham, MA, USA). The supernatant plasma was then transferred to LoBind^®^ Eppendorf tubes and stored at −80 °C until analysis.

### 2.3. Chemicals

AEA (95 µg/mL in ethanol), 2-AG (9500 µg/mL in acetonitrile) and their respective internal standards (AEA-d_4_ in ethanol and 2-AG-d_5_ in acetonitrile, both 1 mg/mL) were supplied by Cayman Chemical (Ann Arbor, MI, USA). Acetonitrile and formic acid were provided by Honeywell (Morristown, NJ, USA), hexane and ethyl acetate by VWR (Radnor, PA, USA), and ultra-pure water was purchased from Merck Millipore (Darmstadt, Germany). Fatty acid-free human serum albumin (HSA, lyophilized powder, ≥99%) was obtained from Sigma-Aldrich (St. Louis, MO, USA) and isotonic Elo-Mel^®^ solution from Fresenius Kabi (Graz, Austria).

### 2.4. Endocannabinoid Measurement

A recently implemented and validated UHPLC-MS/MS method for the quantification of AEA and 2-AG [26] was transferred to a Shimadzu 8060 LCMS system and applied to measure the plasma AEA and 2-AG concentrations.

### 2.5. Questionnaire and Statistical Analysis

The general mood of the participants was assessed immediately pre- and immediately post-run (prior to each blood sampling) by using an 11-point numeric rating scale that asked participants to rate their response to the question “How is your general mood at the moment?” on a scale from zero (worst possible mood) to ten (best possible mood) [27,28,29,30]. This 11-point numeric rating scale expands the rating used, e.g., in the Profile of Mood States (POMS) scale from 5 to 11 points because an 11-point scale has been demonstrated to be the most valid and reliable scale of all the scales included in the study [30]. GraphPad Prism (version 10.1.2.) was used for statistics, and results were generated by applying the nonparametric Wilcoxon signed-rank test as well as the Mann–Whitney U test. Due to the small sample size in the occasional runner’s subgroup in this study, some statistical analyses could not be computed.

## 3. Results

### 3.1. Pre- and Post-Run Mood and Mood Increase Considering Sex, Running Frequency, and Age

**Figure 1:**
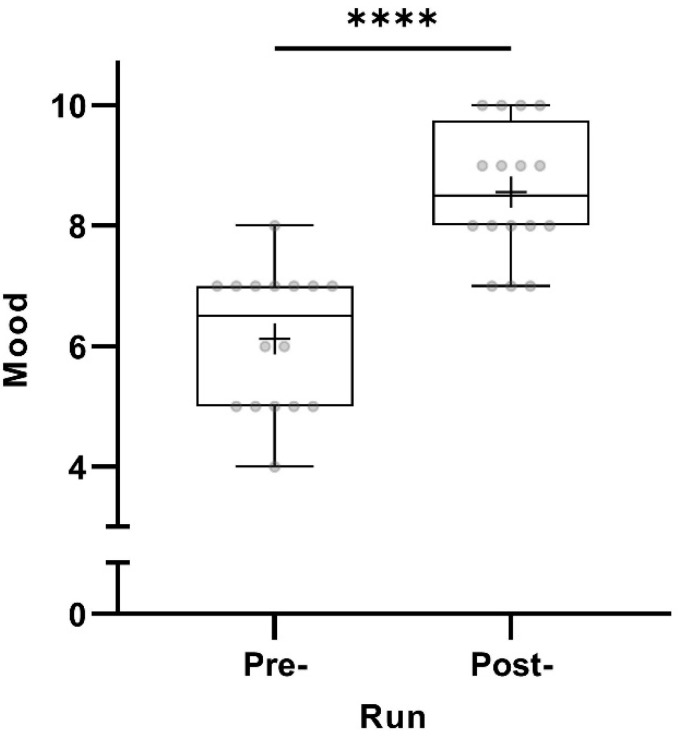
Pre- and post-run mood (in points from 0 to 10) taking all participants into account (n = 16). The horizontal lines in the box depict the median value; the upper and lower horizontal lines represent the interquartile range from quartile 1 to quartile 3; whiskers indicate minimum and maximum values; circles indicate individual values; the overall mean value is indicated by +; **** *p* < 0.0001.

All 16 participants scored their mood as significantly better post-run (*p* < 0.0001) (Figure 1).

**Figure 2:**
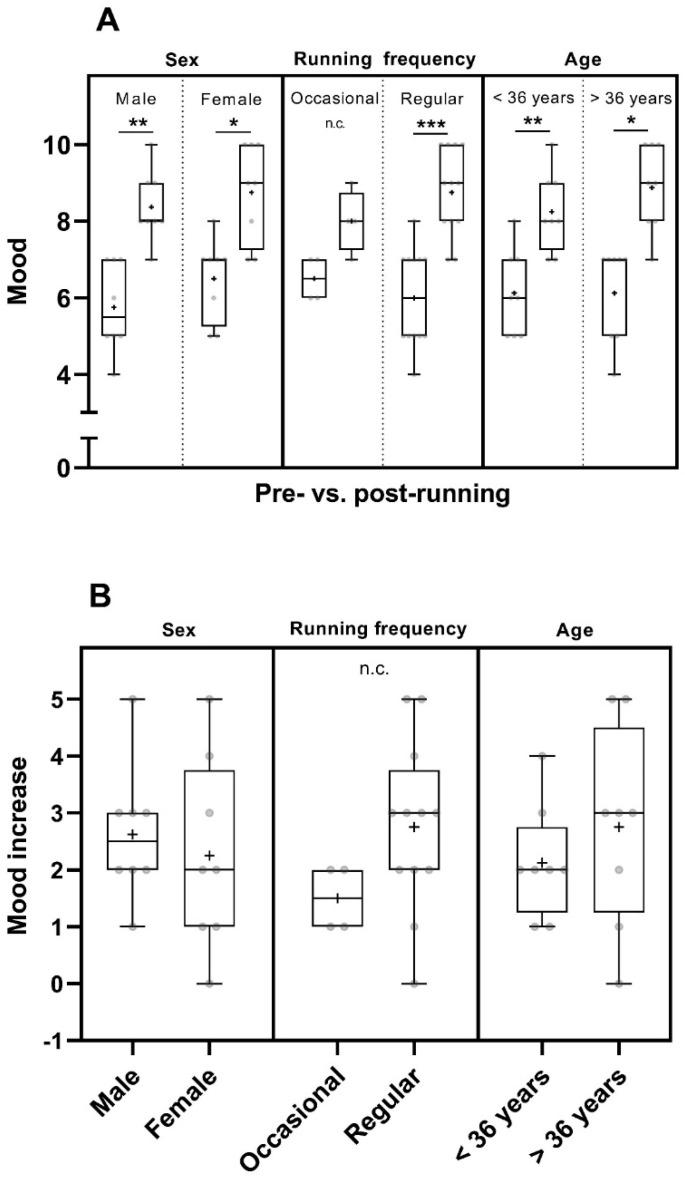
Pre- and post-run mood (in points from 0 to 10) (**A**) and mood increase (in point increase relative to the pre-run mood) (**B**) considering sex [male participants (n = 8) and female participants (n = 8)], running frequency [occasional runners (n = 4) and regular runners (n = 12)] and age [participants < 36 years (n = 8) and participants > 36 years (n = 8)]. The horizontal lines in the box depict the median value; the upper and lower horizontal lines represent the interquartile range from quartile 1 to quartile 3; whiskers indicate minimum and maximum values; circles indicate individual values; the overall mean value is indicated by +; *: *p* < 0.05; **: *p* < 0.01; ***: *p* < 0.001; n.c.: not computed.

Considering the factor of sex, the mood was increased in men (*p* < 0.01) and in women (*p* < 0.05) pre- to post-run. The mean pre- and post-run mood values of women were higher than those of men (Figure 2A and Table S1). When taking the running frequency into account, the post–run mood was significantly increased in participants of the regular runners group (n = 12, *p* < 0.001) (Figure 2A). Data showed a trend towards an increase in post-run mood in occasional runners (Figure 2) but they were not tested statistically due to the small number of participants in this group (n = 4). However, they experienced a trend of higher mean mood increase than that reported by participants of the occasional runner´s group and had a higher mean post-run mood (Figure 2 and Table S1). The mood increased significantly pre- to post-run in participants of both age groups (<36 years: *p* < 0.01; >36 years: *p* < 0.05) (Figure 2A).

### 3.2. Pre- and Post-Run AEA and 2-AG Concentrations and Increase in AEA and 2-AG Concentrations Considering Sex, Running Frequency, and Age

**Figure 3:**
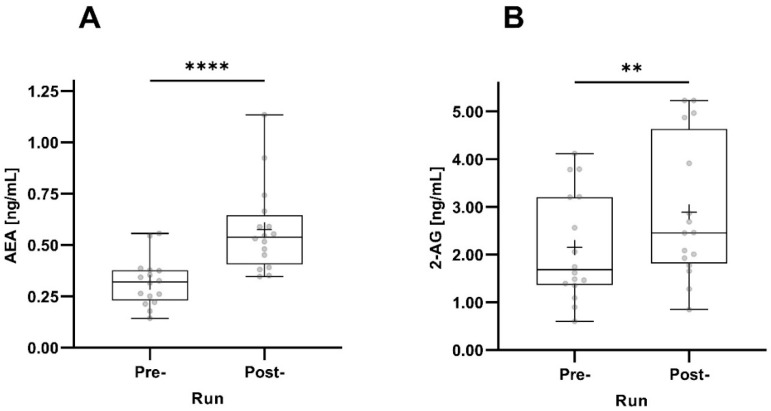
Pre- and post-run AEA (**A**) and 2-AG (**B**) concentrations taking all participants into account (n = 16). The horizontal depicts the median value; the upper and lower horizontal lines represent the interquartile range from quartile 1 to quartile 3; minimum and maximum values are marked by whiskers; circles indicate individual values; the mean value is indicated by +; **: *p* < 0.01; ****: *p* < 0.0001.

Both AEA and 2-AG concentrations significantly increased pre- to post-run. The increase in AEA concentrations was much more pronounced (*p* < 0.0001) than the increase in 2-AG concentrations (Figure 3).

**Figure 4:**
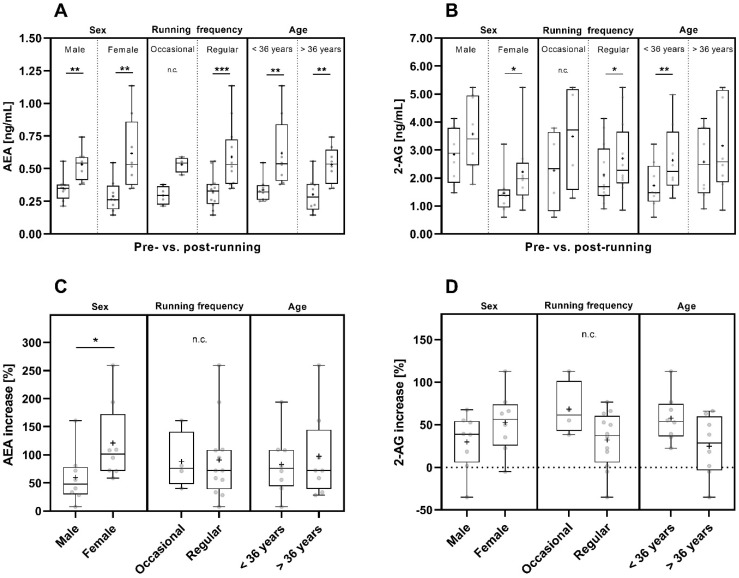
Pre- and post-run AEA (**A**) and 2-AG (**B**) concentrations and their pre- to post-run concentration increases (percent increase compared to pre-run concentrations) (**C**,**D**) considering sex [male (n = 8) and female (n = 8)], running frequency [occasional (n = 4) and regular runners (n = 12)] and age [<36 years (n = 8) and >36 years (n = 8)]. The horizontal line that splits the box into two parts depicts the median value; the upper and lower horizontal lines represent the interquartile range from quartile 1 to quartile 3; minimum and maximum values are marked by whiskers; the mean value is indicated by +; *: *p* < 0.05; **: *p* < 0.01; ***: *p* < 0.001; n.c.: not computed.

Taking sex into account, the mean pre- and post-run AEA concentrations were comparable in male and female participants (Figure 4A and Table S1). However, the post-run AEA concentrations of women spanned a wider range compared to those of men and compared to the pre-run concentrations of 2-AG in men and women (Figure 4A).

The concentrations of both endocannabinoids increased significantly pre- to post-run in female participants (AEA: *p* < 0.01; 2-AG: *p* < 0.05) (Figure 4A,B). Starting from slightly lower mean AEA concentrations pre-run (Figure 4A and Table S1), in female participants the pre- to post-run increase in AEA concentrations was significantly more pronounced than in male participants (*p* < 0.05), (Figure 4C).

In male participants, the pre- to post-run increase in AEA concentrations was also significant (*p* < 0.01), while 2-AG concentrations showed only a trend of increasing values pre- to post-run (Figure 4A,B). 2-AG concentrations showed a greater variation in men than in women (Figure 4B and Table S1). The mean pre- and post-run 2-AG concentrations were nearly two times higher in male (pre-run: 2.843 ng/mL; post-run: 3.569 ng/mL) than in female participants (pre-run: 1.458 ng/mL; post-run: 2.218 ng/mL) (Figure 4B and Table S1).

Considering running frequency, the mean pre- and post-run AEA concentrations were comparable in both groups (Figure 4A and Table S1). However, due to the uneven distribution of participants in the two subgroups and the resulting small number of occasional runners (regular runner: n = 12 and occasional runners: n = 4) the informative value of the calculated statistics for the occasional runners is limited.

Regular runners showed a significant pre- to post-run increase in both AEA and 2-AG concentrations (AEA: *p* < 0.001, 2-AG: *p* < 0.05) (Figure 4A,B). For occasional runners, the significance of the pre- to post-run concentration increase was not computed due to the small number of four runners in this group but there was a trend towards post-run increase in AEA and 2-AG concentrations (Figure 4A,B). Nevertheless, the pre- to post-run increase in 2-AG concentrations had a tendency to be more pronounced in occasional runners than in regular runners (Figure 4D).

Taking the age of the participants into account, the pre- to post-run AEA concentration increase was statistically significant for participants of both age groups (*p* < 0.01), (Figure 4A), and the mean AEA pre- and post-run concentrations were comparable for participants of both age groups (Figure 4A and Table S1). The pre- to post-run increase in 2-AG concentrations was statistically significant in participants below 36 years of age (*p* < 0.01) and was observed as a trend for the participants above 36 years of age (Figure 4B).

## 4. Discussion

The data generated in this study show for the first time the effects of a runner’s high on mood and endocannabinoid concentrations after a 60 min outdoor run in healthy humans considering sex, running frequency, and the age of the study participants.

The mood of all participants was increased significantly post-run which is in line with the well-acknowledged notion that physical exercise has a beneficial impact on mood, feelings, and other effects mediated by the central nervous system [2,3,25,31]. The post-run mood increase observed in our study was accompanied by a highly significant pre- to post-run increase in AEA concentrations, and also by a significant increase in the 2-AG concentrations when considering all participants. Our outdoor results are in line with results from a recent study where increased AEA and 2-AG concentrations as well as euphoria have been detected after a 45 min run on a treadmill [7]. Another study has also found a positive correlation between the exercise-induced increase in AEA concentrations and mood changes [25]. The same study, however, did not find a post-exercise increase in 2-AG concentrations [25]. Other studies have reported a trend of increased AEA concentrations post-physical exercise [16] and also a significant pre- to post-exercise increase in AEA concentrations, but no change in the mood of healthy participants [32]. The reported differences in mood changes post-physical exercise could be partly due to the different measures used to assess the mood, and the subjective nature of the mood experience itself. The numerical rating scale from zero to ten which was used in the present study is a widely used patient-reported outcome scale. It is most commonly used in pain assessment (0: no pain, 10: most intense pain) featuring good reliability in individuals with chronic pain [28,29].

The highly significant post-run increase in AEA concentrations in our outdoor study matches the increased AEA concentrations after a single episode of 30 to 90 min indoor training observed in healthy participants in previous studies [15,17,25,32,33]. Also, well-trained healthy participants have exhibited significant increases in AEA concentrations after outdoor physical exercise, namely hiking [21]. There is only one previous study where AEA concentrations have not been increased post-exercise, but these results have only been found in participants who were exercise-addicted [32].

Although, 2-AG concentrations were increased after a 60 min outdoor run in our study, data had a larger variation than AEA data which corresponds to previously reported varying effects of physical exercise on 2-AG concentrations: Some previous studies have observed stable 2-AG concentrations pre- and directly post-physical exercise [15,21,25,33], while AEA concentrations were always significantly increased post-physical exercise [17,25,33]. Significantly increased 2-AG concentrations have also been reported after three minutes of submaximal static muscle contractions of the forearm [34]. In contrast, the authors of another study have subjected healthy young men to 80 days of training with two-thirds moderate-intensity and one-third high-intensity training and significantly decreased post-exercise 2-AG concentrations have been reported [35].

Altogether, these findings indicate that although AEA concentrations seem to be increased post-exercise, the direction of 2-AG concentration changes remains unclear. The significant decrease in 2-AG concentrations post-regular physical exercise [35] could also signify an adaptive response linked to improved fitness.

Mood and endocannabinoid concentration changes post-physical exercise have never been investigated considering demographic parameters such as sex and age, as well as the running frequency of the participants. We found significantly increased mood and also significantly increased AEA concentrations pre- to post-run in all three groups allocated by sex, running frequency, and age; with the exception of the group allocated by running frequency where the small number of participants (four occasional runners) did not allow for meaningful statistical testing. However, we observed a trend of increased AEA concentrations post-run in occasional runners. In contrast to post-run AEA concentration changes, the post-run 2-AG concentration changes were only significant for female participants, those of the regular runners group, and those below 36 years.

Sex-related differences in mood changes and endocannabinoid concentration changes post-run were observed in our study. AEA concentrations increased to a greater extent in female participants than in male ones which is in agreement with results from a previous animal study [36]. Notably and contrary to AEA, absolute 2-AG concentrations were almost two-fold lower in females than males both pre- and post-run, but relative 2-AG concentrations increases were comparable in both sexes. The higher absolute values of mood reported by female participants both pre- as well as post-run can underline the assumption that sex hormones regulate receptors and enzymes of the endocannabinoid system and can therefore influence endocannabinoid concentrations depending on the subject’s sex [37].

Taking the running frequency into account, the pre- to post-run mood increase was significant in participants of the regular runners group and presented as a trend in those of the occasional runners group. The relation between mood change post-physical exercise and the frequency of physical exercise has rarely been studied until now. Our results suggest that individuals who are running more frequently can experience a more pronounced mood elevation post-physical exercise. This has also been observed in a previous study in which mood improvement but no change in endocannabinoid concentrations have been reported in exercise-addicted participants post-physical exercise [32]. However, in order to be able to perform comprehensive and reliable investigations on the correlation between running frequency and mood, it will be necessary to perform additional studies with a further refined classification regarding running frequency.

In our study, occasional runners had more pronounced pre- to post-run 2-AG concentration increases than regular runners; an effect which was not observed in AEA concentration increases pre- to post-run. However, regular runners experienced a stronger mood increase and a better post-run mood than occasional runners. These different responses in AEA and 2-AG concentration increases between regular and occasional runners might further indicate that regular physical exercise may lead to an adaptive response linked to improved fitness with decreased 2-AG but not AEA concentrations. The smaller post-run increase in 2-AG concentrations in regular runners is consistent with the observations of Koay et al. who have reported a significant decrease in 2-AG concentrations in healthy participants engaged in a long-term training program [35].

We further considered the influence of age on runner’s high in our study. While participants from both age groups experienced comparable pre-run mood, those older than 36 years had a more increased mean post-run mood than the younger participants. 2-AG concentrations increased pre- to post-run to a smaller extent in the older than in the younger participants. However, these results might carry a certain degree of bias as the participants in the two age groups were unevenly distributed: participants above 36 years were predominantly regular runners (seven regular and one occasional runner) whereas the participants below 36 years comprised five regular and three occasional runners. This distribution has also influenced the finding of a smaller increase in 2-AG concentrations in regular relative to occasional runners. Nevertheless, our results suggest that age can modulate endocannabinoid responses during an endurance outdoor run and may thereby influence mood changes, but further investigations with more even group distributions are needed.

This is the first study that investigated the effects of a runner’s high after a 60 min outdoor run. There is only one other study that has investigated the effects of physical exercise on endocannabinoid concentrations under outdoor conditions. However, the participants in this previous study did not perform an endurance run of one hour but had been hiking for several hours [21]. The different responses to physical exercise in terms of the endocannabinoid concentrations and mood of the participants in our study may also be due to our study design including non-standardized experimental conditions such as the presence of external confounding factors. However, we chose this study design to promote a runner’s high, as the highly standardized laboratory environment can also be detrimental to the development of a runner’s high. Siebers et al. have observed that only few people who had already felt a runner’s high have also been able to experience this phenomenon under laboratory conditions [7].

One limitation of this study is the small overall sample size (n = 16) and especially the small sample size of the subgroups. In order to show more statistically verified effects of physical exercise on the endocannabinoid concentrations taking sex, running frequency, and age of the participants into account, a larger study is needed. The results generated in this study, however, can serve as preliminary results to estimate the effects of runner’s high in the specific subgroups. Another general limitation of this study is that mood is a subjective feeling and can hardly be objectively assessed, but the numeric rating scale we have used is acknowledged as a standard method with very high reliability [28,29].

## 5. Conclusions

The data showed the impact of an outdoor run on mood and endocannabinoid concentrations considering sex, running frequency, and age. We found significant post-run increases in mood and AEA and 2-AG concentrations when considering all participants. 2-AG concentrations had a higher variation than AEA concentrations, which is in line with the already reported inconsistent relation between physical exercise and 2-AG concentration changes. Our data showed notable differences in post-run AEA concentrations increases regarding sex, and trends when regarding running frequency and age.

Outcomes from this study underscore the connection of physiological and psychological variables during runner’s high and the observed patterns regarding the interplay of mood and endocannabinoid concentrations offer promising avenues for further investigation and provide a solid foundation for future research on human runner’s high. Nevertheless, future studies on runner’s high are needed, including monitoring of heart rate and exercise intensity, to better control the intensity of exercise for each participant and thus reduce the variability of responses to exercise.

## Data Availability

The data presented in this study are available on request from the corresponding author.

## Supplementary Materials

The following supporting information can be downloaded at: https://www.mdpi.com/article/10.3390/sports12090232/s1, Table S1: Pre- and post-run mean, minimum, and maximum values for mood, AEA, and 2-AG concentrations.
