# Supplementary material for: Investigating Runner’s High: Changes in Mood and Endocannabinoid Concentrations after a 60 min Outdoor Run Considering Sex, Running Frequency, and Age
Source: Sports (Basel). 2024 Aug 28;12(9):232. doi: 10.3390/sports12090232 (PMC11435531; doi:10.3390/sports12090232)
Supplement: Supplementary file 1 [file sports-12-00232-s001.zip › sports-3111889-supplementary.pdf]

SUPPLEMENTARY MATERIAL

Table S1: Pre- and post-run mean, minimum, and maximum values for mood, AEA, and 2-AG concentrations taking the groups sex, running frequency, and age into account.

			Mood								
			pre-run				*post-run*				Abs. diff.
Groups	Subgroups	n	Min	Max	Mean	SD	Min	Max	Mean	SD	
**Sex**	Female	8	5	8	6.500	1.069	7	10	8.750	1.282	2.250
	Male	8	4	7	5.750	1.165	7	10	8.375	0.916	2.625
**Running frequency**	Occasional	4	6	7	6.500	0.577	7	9	8.000	0.816	1.500
	Regular	12	4	8	6.000	1.279	7	10	8.750	1.138	2.750
**Age**	< 36 years	8	5	8	6.125	1.126	7	10	8.250	1.035	2.125
	> 36 years	8	4	7	7.000	1.246	7	10	9.000	1.126	2.000

			AEA								
			pre-run				*post-run*				Abs. diff. [ng/mL]
Groups	Subgroups	n	Min conc. [ng/mL]	Max conc. [ng/mL]	Mean conc. [ng/mL]	SD	Min conc. [ng/mL]	Max conc. [ng/mL]	Mean conc. [ng/mL]	SD	
**Sex**	Female	8	0.144	0.545	0.289	0.128	0.347	1.134	0.617	0.280	0.327
	Male	8	0.213	0.557	0.350	0.102	0.381	0.742	0.532	0.117	0.183
**Running frequency**	Occasional	4	0.213	0.378	0.295	0.072	0.451	0.589	0.531	0.059	0.236
	Regular	12	0.144	0.557	0.327	0.124	0.347	1.134	0.589	0.233	0.261
**Age**	< 36 years	8	0.251	0.545	0.337	0.096	0.381	1.134	0.618	0.270	0.281
	> 36 years	8	0.144	0.557	0.302	0.138	0.347	0.742	0.531	0.139	0.229

			2-AG								
			pre-run				*post-run*				Abs. diff. [ng/mL]
Groups	Subgroups	n	Min conc. [ng/mL]	Max conc. [ng/mL]	Mean conc. [ng/mL]	SD	Min conc. [ng/mL]	Max conc. [ng/mL]	Mean conc. [ng/mL]	SD	
**Sex**	Female	8	0.602	3.213	1.458	0.784	0.851	5.235	2.218	1.338	0.760
	Male	8	1.466	4.123	2.843	1.024	1.776	5.234	3.569	1.348	0.727
**Running frequency**	Occasional	4	0.602	3.784	2.264	1.483	1.280	5.234	3.484	1.930	1.220
	Regular	12	0.897	4.123	2.112	1.072	0.851	5.235	2.697	1.332	0.584
**Age**	< 36 years	8	0.602	3.206	1.729	0.839	1.280	4.968	2.635	1.245	0.906
	> 36 years	8	0.897	4.123	2.571	1.285	0.851	5.235	3.152	1.718	0.581

SD = Standard deviation, Conc. = Concentration, Abs. diff. = Absolute difference
